# Extracts from an Address Delivered in Chicago December 10, 1915, before the Eugenics Educational Society

**Published:** 1916-02

**Authors:** Casper L. Redfield


### The Department of Eugenics, Mental and Social Hygiene

CONDUCTED BY
DR. MALONE DUGGAN
813-814 Gibbs Building, San Antonio, Texas
All communications for this department should be sent to DR. DUGGAN

### Extracts From an Address Delivered in Chicago December 10, 1915, Before the Eugenics Educational Society.

BY CASPER L. REDFIELD.

It has been said frequently that each man is the product of his environment. In a measure that is true, but no environment will make a Shakespeare out of an ordinary man. A great man is born, which means that he is the product of a particular kind of breeding. Similarly, the feeble-minded man is born, which also means that he is the product of a particular kind of breeding. The kinds of breeding which produce our great men and our feeble-minded men are as widely separated as are the men themselves. I cannot, in the time at my disposal, go into all of the intricacies by which different kinds of men are produced by breeding, but I can give some of the main essentials, and from these you can obtain a fairly clear understanding of what it is that leads toward improvement, and what it is that leads toward degeneracy.

They tell us that man and the other higher animals have evolved from lower forms of animals by selection, but those who make that statement overlook a very obvious absurdity in their claim. To have selection, parents must have offspring, and to have more selection the offspring must produce another generation, and these in turn another. Each generation gives opportunity for selection, and the more generations the more selection. Anything which would reduce the number of generations in a given period of time would reduce the opportunities for selection to accomplish anything.

At some time in the past there was a common ancestor for man and the higher apes. There have been less generations, and consequently less selection, in the line leading from that common ancestor to man, than in the lines leading to the apes. Further back in the past there was a common ancestor for the higher apes and the lower monkeys. There have been less generations, and consequently less selection in the lines leading to the higher apes than in the lines leading to the lower monkeys. Extend that examination to the different species of active animals and you will find that each advance from a lower to a higher stage involved the elimination of selection, and that the actual advance has been inversely proportional to the amount of selection. Carried to its logical conclusion this means that the greatest possible advance will occur when selection is reduced to zero.

They tell us that acquired characters are not inherited, but I am telling you that the persons that make that statement never investigated the matter and know nothing whatever about it. They simply repeat what they have been taught, and they cling to the dogma because it agrees with their preconceived ideas. The theory that acquired characters are not inherited originated in a misconception of what an acquired character is, and in an experiment which is absurd on its face.

To acquire means to obtain by effort, by exertion, by the performance of work. An acquired character is one obtained by exercising an organ, or by the work performed by the organ. It consists of a physiological change occurring within the organ which is dynamic in character and is called dynamic development. The amount of an acquirement is proportional to the amount of work performed. A mentally active man has a better developed brain at the age of 50 than he had at the age of 20, and the difference is due to the extra amount of mental work performed.

If an acquirement is to be inherited, the parent must make the acquirement first and get the offspring afterwards, not get the offspring first and make the acquirement afterwards. Of those who deny the inheritance of acquired characters, what one ever took this into consideration and compared the progeny of parents of different ages on the basis of acquirement? Not one. They have failed to take even the first step in such an investigation, whereas they should carry such a one through three or four generations of ancestors.

Among certain eugenists there is a theory that it is impossible to produce an individual which is superior to anything which previously existed. That is, if some very superior individual exists it is because there was, somewhere in his ancestry, a similar superior individual. This theory amounts to a denial of evolution and a return to the Garden of Eden story with Adam and Eve originally created equal to any individual who has since existed.

It is not clear how widely extended this theory is, but it seems to be back of the proposition to sterilize a large part of the population. That proposition is a public confession, by those who make it, that they know absolutely nothing about what causes improvement and what causes degeneracy. In their despair at seeing no way to improve the race other than that of killing off the inferior, they propose the killing process by indirection.

It seems never to have occurred to these gentlemen to write out the pedigree of some remarkable individual for three or four generations and then examine that pedigree for the purpose of learning if there was anything remarkable about the way he was produced. Wedded to a preconceived theory which they are anxious to support, they make statements without stopping to consider what those statements mean when carried to their logical conclusion.

Let us consider the horse. A century ago there was no horse in the world capable of trotting a mile in three minutes. Now we have horses which have trotted a mile in two minutes. This an absolute and very great advance in power made in the past one hundred years. It has been said repeatedly that this improvement came about through selection, but the statement is not true and it is made in complete ignorance of the facts. Selection has been used abundantly among horses, but that selection is not connected with the improvement which has taken place.

High speed at the trot is not a natural gait for horses. It is an artificial gait which never existed in any breed of horses until forced there by the art of man. Less than a century ago the only high speed gait for horses was the run, and when trotters were forced for speed they would break into a run. Now we have "born trotters" which will stick to the trot no matter how hard they are forced, and trotting speed approaches running speed. Here is a new character in the trotter of today.

To have selection a mare must have several foals. If she produces but one foal in her entire life, there can be no selection in her line. It is take that foal or none. Write the pedigree of any 2:10 trotter, it matters not what one, and extend that pedigree to the time when there was no such thing in the world as a 2:30 trotter. In that pedigree there will be from five to twenty mares, no one of which ever had more than one foal in her life. The other mares, and the sires in the pedigrees, will be found, on investigation, to have produced less than the normal number of foals.

Also, the lines of improvement to our high speed trotters average only seven generations to the century, while the normal number is ten generations. Actual improvement came in those lines in which opportunity for selection was reduced to its lowest limit.

The same thing is true in intellectual power in man. Take any list of intellectually eminent men and you will find that they were sons of men much older than the average age of fathers when sons are born. A few will be found to be sons of comparatively young fathers, but push the investigation in those cases a little further and you will find that while it is possible to get an eminent man from a young father it is impossible to get one from a succession of young parents. A succession of young parents always results in the production of mental inferiority, and, if the parents are unusually young, in such a succession the product is weak mentality.

To maintain any group of animals on a level in its power capabilities there must be a certain amount of acquirement per generation before reproduction. If the amount of acquirement is decreased there is a decline in power capabilities toward a lower corresponding level. If the acquirement is increased there is a rise toward a higher corresponding level. The age of parents at time of reproducing is one factor in measuring the amount of acquirement, and an investigation which did not consider this factor in at least three generations of ancestors would be superficial.

